# Optimizing Appearance-Based Localization with Catadioptric Cameras: Small-Footprint Models for Real-Time Inference on Edge Devices

**DOI:** 10.3390/s23146485

**Published:** 2023-07-18

**Authors:** Marta Rostkowska, Piotr Skrzypczyński

**Affiliations:** Institute of Robotics and Machine Intelligence, Poznan University of Technology, 60-965 Poznan, Poland; piotr.skrzypczynski@put.poznan.pl

**Keywords:** omnidirectional vision, mobile robot, localization, deep learning, edge computing

## Abstract

This paper considers the task of appearance-based localization: visual place recognition from omnidirectional images obtained from catadioptric cameras. The focus is on designing an efficient neural network architecture that accurately and reliably recognizes indoor scenes on distorted images from a catadioptric camera, even in self-similar environments with few discernible features. As the target application is the global localization of a low-cost service mobile robot, the proposed solutions are optimized toward being small-footprint models that provide real-time inference on edge devices, such as Nvidia Jetson. We compare several design choices for the neural network-based architecture of the localization system and then demonstrate that the best results are achieved with embeddings (global descriptors) yielded by exploiting transfer learning and fine tuning on a limited number of catadioptric images. We test our solutions on two small-scale datasets collected using different catadioptric cameras in the same office building. Next, we compare the performance of our system to state-of-the-art visual place recognition systems on the publicly available COLD Freiburg and Saarbrücken datasets that contain images collected under different lighting conditions. Our system compares favourably to the competitors both in terms of the accuracy of place recognition and the inference time, providing a cost- and energy-efficient means of appearance-based localization for an indoor service robot.

## 1. Introduction

The rapid development of robotics and artificial intelligence applications is leading to the proliferation of mobile service robots [1,2]. Technological advancements, such as artificial intelligence and machine learning, have significantly improved the capabilities and autonomy of these robots, making them more efficient and reliable in performing various tasks. Additionally, the increasing demand for automation and efficiency in industries such as healthcare, hospitality, and logistics has created a strong market incentive for developing and deploying service mobile robots.

Also, the growing need for eldercare robots has become increasingly evident as the global population ages. These robots can provide valuable assistance and companionship to older adults, monitoring their health and enhancing their overall well-being [3]. However, these robots must be affordable to ensure widespread accessibility and adoption among families and caregivers [4].

A common requirement in these service robots is to be able to localize within their workspace, which is usually a man-made indoor environment [5]. Although precise position tracking can be provided by a SLAM (simultaneous localization and mapping) system using vision or RGB-D data, the issue of global localization remains a problem when the robot’s previous position data cannot be used [6]. Such a problem in practice arises, for example, in dynamic environments due to occlusions. There are practical global localization algorithms, such as the one proposed in our previous work [7], but they have two functional limitations, namely, they require long-range sensors to extract features that are distant from the robot and are computationally expensive. These features make them unsuitable for a small and inexpensive service robot.

**Figure 1:**
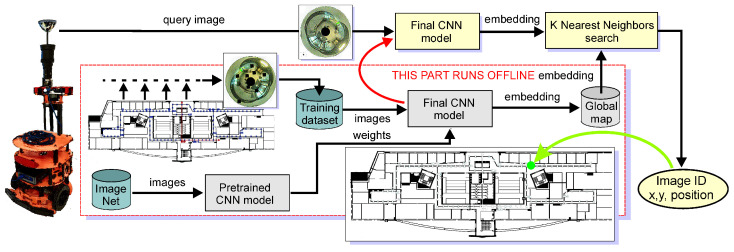
Overview of the proposed method—a flowchart of the appearance-based localization system. The service robot is shown with the latest, larger-field-of-view catadioptric camera, but without the perspective camera, which is not used in this research.

Therefore, we propose a solution to the problem of global localization in a known (entirely or partially) environment using a passive catadioptric camera and the principle of recognizing places previously visited by the robot (Figure 1). The applied sensor with a catadioptric camera is a variant of the biologically inspired sensor with a hybrid field of view that we introduced in [8]. This sensor uses a catadioptric camera to achieve omnidirectional vision, an analogue of the peripheral vision found in vertebrates [9]. It allows animals to orient themselves to changes and hazards in the environment quickly. The sensor described in [8,9] is complemented by a moving perspective camera that performs the functions of foveal vision, the more accurate but spatially limited vision mode in animals. This function is not used in the research presented in this article, as we limit the scope to global appearance-based localization, i.e., the assignment of the robot’s current location to one of the previously recognized (visited) places. Our approach yields information about the similarity of the places observed in the current perception and locations stored in a reference map.

Although appearance-based localization does not provide an accurate metric position of the robot in a global reference system, the ability to tell if the robot is close to one of the known locations is often sufficient for indoor navigation [10]. If a map of reference places is collected at high density (e.g., based on a grid with cells of one meter in size or smaller), this kind of localization may be sufficient for the service robot’s tasks. In addition, appearance-based localization can be supplemented by visual odometry or the recognition of artificial landmarks deployed at a given location [11]. The perspective camera of a hybrid sensor can be used to perform these functions. The main objectives of this research work are the following:

- Experimental analysis of neural network architectures in search of an architecture for an image-based place recognition system suitable for implementation on an embedded computer of an intelligent vision sensor with limited power and resources.
- Experimental verification of the possibility of using catadioptric camera images in the appearance-based localization task without developing them into panoramic form significantly reduces the computational load.
- Analysis of the strategy for creating training sets in a place recognition task, assuming that the obtained solution should be generalized to different image acquisition conditions, mainly depending on illumination.

We propose a novel approach that adopts a convolutional neural network (CNN) architecture to directly process the omnidirectional images for real-time place recognition to meet these objectives. CNNs are specialized for processing grid-like data, particularly images, using convolutional layers and parameter sharing to capture spatial patterns effectively. The proposed system leverages the concept of global image descriptors, which are already proven to be efficient in place recognition [12]. We employ a CNN to produce the descriptors in the form of embedding vectors directly from the omnidirectional images, thus avoiding the processing overhead required for computing undistorted panoramic images, which are often used in appearance-based localization with catadioptric cameras [13]. The proposed architecture is optimized for inference on the Nvidia Jetson TX2 edge computing platform integrated with our sensor. The low-cost Jetson TX2 board is designed for peak processing efficiency at only 7.5 W power. Regarding energy consumption for image processing, the Jetson TX2 has a clear advantage over an x86-based platform [14]. While the exact power consumption will depend on the specific image processing workload, the Jetson TX2 is designed to provide a good balance between performance and energy efficiency [15]. Hence, by applying an integrated sensor with an edge computing platform and developing a matching small-footprint neural network architecture, we obtain a self-contained, energy-efficient, and compact system for real-time appearance-based localization that can be integrated with practically any mobile service robot, providing this robot with reliable global localization capabilities at low cost. The contribution of this paper is threefold:

1. A novel, simple-yet-efficient CNN-based architecture of the appearance-based localization system that leverages a lightweight CNN backbone trained to apply transfer learning to produce the embeddings and the K-nearest neighbours method for quickly finding an embedding matching the current perception.
2. A thorough experimental investigation of this architecture, considering several backbone network candidates and omnidirectional or panoramic images used to produce the embeddings. The experiments were conducted on three different datasets: two collected with variants of our bioinspired sensor and one publicly available.
3. An investigation of the strategies for creating the training set and the reference map for the localization system conducted on the COLD Freiburg dataset. This part of our research allowed us to test how our neural network model generalizes to images acquired under different lighting/weather conditions. It resulted in the recommendation of using data balanced concerning their acquisition parameters, improving generalization.

The remainder of this article is structured as follows. Most important related works are reviewed in Section 2. Section 3 introduces the proposed architecture of the localization system and details the neural networks being used. Next, Section 4 describes the experimental setups and dataset used to test various aspects of the proposed solution, while Section 5 provides the results of experiments and contributes an in-depth analysis of the performance of different variants of the investigated system. Finally, Section 6 concludes the article and proposes future extensions.

## 2. Related Work

Appearance-based localization from omnidirectional images has garnered significant attention in computer vision and robotics. Researchers have developed various techniques to address the challenges posed by the distortion and wide field of view of omnidirectional cameras. This section reviews the most relevant works that have contributed to the state of the art in this area.

The application of passive vision sensors for localization was extensively researched in robotics, resulting in several visual Simultaneous Localization and Mapping (SLAM) algorithms [16]. However, the applications of visual SLAM on commercially viable mobile robots are limited by the often-insufficient on-board computing resources of such robotic platforms and due to problems raised by the changing lighting conditions, rapid changes of viewpoint while the robot is moving, and the lack of salient local features in some indoor environments. Moreover, SLAM does not guarantee to solve the global localization problem whenever the robot loses track of its pose due to any of the issues mentioned above [17].

Therefore, the appearance-based recognition of locations becomes an exciting addition to visual SLAM for closing the loops and relocalizing a lost robot [18]. This approach, in many variants, is also considered a localization method on its own, which is particularly suitable for large-scale outdoor scenarios [10]. Unlike the visual SLAM algorithms, appearance-based localization methods only determine if the observed scene resembles an already visited location. However, the place recognition methods scale better for large environments than typical SLAM algorithms [19]. In this context, catadioptric cameras yielding omnidirectional images improve the reliability of place recognition for robot localization in comparison to the narrow-field-of-view perspective cameras, as demonstrated by the work on the COsy Localization Database (COLD) dataset [20], which we also use to evaluate our localization system. An interesting research direction is to use image sequences instead of individual images, which decreases the number of false positives in place recognition for environments with self-similarities and increases the robustness of scene dynamics [12]. We applied this idea in our earlier work on place recognition for mobile devices [21], making it possible to implement robust place recognition on a smartphone with very limited computing power, while still using nondistorted perspective images.

In the appearance-based methods, each image is described by descriptors of salient features contained in this image, or is directly described by a whole-image descriptor. Although SURF features were used directly in appearance-based localization performing image retrieval in a hierarchical approach [22], the direct matching of local features is considered inefficient for place recognition [10] if point feature descriptors are used (such as the popular SIFT, SURF, and ORB [23]). Hence, the bag of visual words (BoVW) technique [24] is commonly used, which organizes the features into a visual vocabulary. Next, images described by visual words can be efficiently matched by comparing binary strings or histograms. One prominent example of a location recognition algorithm employing the BoVW technique is FAB-MAP [25,26], which efficiently compares images with a histogram-based approach.

Global image descriptors have proven effective for capturing the overall appearance of omnidirectional images [27]. Earlier works focused on adapting existing, general-purpose feature extraction and matching algorithms. Menegatti et al. [28] proposed using the Fourier transform to handle geometric distortions in catadioptric images. More recently, Payá et al. [29] introduced a method based on the Radon transform to extract global environmental descriptions from omnidirectional images. These works provided foundations for subsequent research by addressing the specific characteristics of omnidirectional images. Examples of hand-crafted descriptors adopted for the global description of omnidirectional images include HOG (histogram of oriented gradients) [30] and Gist [31], which were applied to omnidirectional images from a catadioptric camera in appearance-based localization by Cebollada et al. [32]. While both these methods of image description provided relatively efficient descriptions of the images, allowing the localization system to recognize the places accurately, the descriptor construction algorithms initially developed for perspective camera images required the catadioptric images to be undistorted and converted to panoramic images, which creates a significant computation overhead.

Machine learning methods have gained popularity in place recognition, also from omnidirectional images [33]. Working with typical perspective images, Li et al. [34] proposed an image similarity measurement method based on deep learning, which combines local and global features to describe the image and can be used for indoor place recognition for a robotic agent. Significant progress in appearance-based localization and navigation was achieved by the NetVLAD approach [35], a CNN-based method that aggregates local features for global image representation. The NetVLAD network consists of a CNN for feature extraction and a layer based on vector of locally aggregated descriptors—VLAD [36]. In this architecture, VLAD is a feature quantization technique similar in concept to the bag of visual words idea, as it captures information about the statistics of an image’s local descriptors. The VLAD is a method for combining descriptors for both instance-level searches [37] and image classification [38]. Although Cheng et al. [39] used NetVLAD with panoramic images from an omnidirectional system, this approach was demonstrated successfully, mainly in outdoor scenarios working with perspective camera images. For indoor scenarios, [13] introduced the omnidirectional convolutional neural network (O-CNN) architecture, which, similarly to our approach, is trained to retrieve the closest place example from the map. Whereas the O-CNN architecture takes advantage of the omnidirectional view by incorporating circular padding and rotation invariance, it requires the omnidirectional images to be converted to their panoramic counterparts. Also, Cebollada et al. [40] demonstrated the benefits of solving localization problems as a batch image retrieval problem by comparing descriptors obtained from intermediate layers of a CNN. A CNN processing rectangular panoramic images reconstructed from the original catadioptric input is used in this work.

As the construction of invariant feature descriptors for omnidirectional images is problematic, Masci et al. [41] proposed to learn invariant descriptors with a similarity-preserving hashing framework and a neural network to solve the underlying optimization problem. Ballesta et al. [42] implemented hierarchical localization with omnidirectional images using a CNN trained to solve a classification task for distinguishing between different rooms in the environment and then a CNN trained for regression of the pose within the recognized room. Although this solution does not require converting the catadioptric images into panoramic ones, its performance is limited by the employed two-stage scheme with separated classification and regression steps. More recent work from the same team [43] solved the appearance-based localization problem by applying a hierarchical approach with the AlexNet CNN. Assuming an indoor environment, they first accomplished a room retrieval task and then carried out the fine localization step within the retrieved room. To this end, the CNN was trained to produce a descriptor, which was compared with the visual model of the selected room using a nearest neighbour search. This approach does not require panoramic conversion of the collected catadioptric images and is overall most similar to the solution proposed in this paper. However, we introduce a much simpler, single-stage architecture based on a recent, lightweight CNN backbone, and the concept of direct retrieval of the image stored in the environment map, which is most similar in appearance to the query image. The efficient process of constructing the embeddings from a pretrained CNN, followed by a fast comparison of these embeddings/descriptors in the KNN framework, allowed us to give up with separated room retrieval in favour of a single-stage architecture, which suits our embedded computing platform well. We compare it directly to the results shown in [43] on the COLD Freiburg dataset, demonstrating our approach’s superior performance and real-time capabilities.

## 3. Localization System Architecture

**Figure 2:**
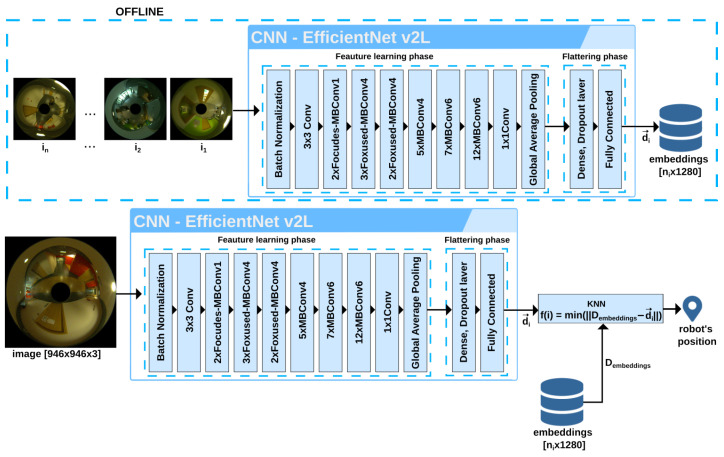
Diagram of the CNN-based image description blocks that produce embeddings used as global descriptors in the localization system. The global map is built from $n_{i}$ images ($i_{1}\ldots i_{n_{i}}$) converted to embedding vectors ${\vec{d}}_{i}$ that are stored in the map $D_{\mathrm{embeddings}}$ of $n_{i}$ embeddings (global descriptors). Note that panoramic images can be used as well instead of the omnidirectional ones.

In the proposed localization system, the robot figures out its current location by determining the similarity between the currently captured image (query image) and images stored in a database (map) describing the environment. This task refers to efficient, real-time image retrieval [10]. The localization procedure involves comparing a global descriptor constructed in real time from the image currently captured by the robot with a previously prepared database of descriptors representing the images of previously visited places and finding the image with the highest possible similarity in the feature space (Figure 2). Each location has its representation in the prepared database of images, and the locations where the images were taken are assumed to cover the entire robot’s workspace. Images from the database are recorded at known locations, so finding one with the minimum distance (in the sense of similarity of appearance) to the current perception allows our robotic agent to approximate its location in the real world.

The proposed localization system uses a CNN to determine the set of natural features for a given location, and the K-nearest neighbours (KNN) [44,45] algorithm to find the closest image from the provided database of images.

CNN and KNN are both machine learning techniques, but differ in their approach and application. CNN learns hierarchical representations of data through multiple convolutional layers, pooling, and fully connected layers. In contrast, KNN is a simple and intuitive algorithm for classification and regression tasks. It makes predictions based on the similarity of new data points to the existing labelled data points in the feature space. One can use CNN to extract features from images and then apply KNN to those extracted features for classification. This hybrid approach leverages the strengths of both algorithms, with CNN capturing intricate patterns and KNN using the extracted features for classification [46]. This idea is used in our localization system. The backbone CNN creates descriptors, which hereinafter are also called “embeddings”, directly from the omnidirectional images, avoiding the additional computations required to obtain undistorted panoramic images. The KNN algorithm uses the embeddings that encode the most salient features of the observed places, to find in the database (i.e., the global map) the images that best match the current observation. In Section 4, we demonstrate that the accuracy of localization with raw catadioptric images is at least as good as with the converted panoramic images, while it demands less computing power.

The preparation of the CNN model is based on training the network to correctly recognize places, with the specific aim of training the higher layers of the network to extract feature maps specific to each location properly. Because the CNN used as the backbone of our system is pretrained on images unrelated to the target domain (ImageNet dataset [47] was used in pretraining), the network was fine-tuned before use by unfreezing several layers and training on the target domain images using cross-entropy as a loss function. Cross-entropy defines the distance between two probability distributions according to the equation:

(1) $$H\left(P\left(y,1-y\right),\;P\left(y_{\mathrm{pred}},1-y_{\mathrm{pred}}\right)\right)\;=\;P\left(y\right)\log\left(P(y_{\mathrm{pred}})\right)+P\left(1-y\right)\log\left(P(1-y_{\mathrm{pred}})\right),$$

where y—the actual location; $y_{\mathrm{pred}}$—the location obtained via a neural network; P(y,1−y)—the probability distribution of the actual location; $P(y_{\mathrm{pred}},1-y_{\mathrm{pred}})$—the probability distribution of the location determined via a neural network (prediction).

At first, the images are processed by the trained convolutional neural network, from which the output layer was removed, to obtain descriptors (in the form of embedding vectors) that describe the global characteristic features of each image in the database, i.e., each unique place visited by the robot. In this way, a global map of all locations based on reference images is created. Not all convolutional network architectures from the literature can be used on a robotic onboard computer with fewer computational and memory resources.

This research uses backbone networks from the MobileNet [48] and EfficientNet [49] families, which are optimized for mobile devices while ensuring high accuracy with a minimal number of parameters and mathematical operations. The MobileNet model uses depth-separated convolution layers consisting of depth-wise convolution and point-wise convolution. Convolution concerning depth (spatial convolution) is used to apply a single filter for each input channel. In MobileNet V2, a new module with inverted residual structure has been introduced, there are two types of blocks. One is an inverted residual block of width 1. The other one is a block of width 2 to reduce the size of the feature map. There are three layers for both types of blocks. The first layer is a 1 × 1 convolution with the ReLU activation function, and the second layer is a convolution against depth. The third and final layer is another convolution of size 1 × 1, with linear bottlenecks. Residual blocks connect the beginning and end of the convolutional block via a skip connection. Adding these two states allows the network to access previous activations not modified in the convolution block. This approach has proven to be essential for building networks of large depths. In MobileNet V2, the basic convolutional layer is called MBConv and contains an inverted residual block with linear bottleneck and depth-separated convolution, with batch normalization behind each convolutional layer.

The EfficientNet model, which we have selected for our final architecture, can be seen as a further step towards efficiency compared to the MobileNet model. EfficientNet uses a complex model scaling technique based on a set of specified coefficients. Instead of randomly scaling width, depth, or resolution, compound scaling uniformly scales each dimension using some fixed scaling coefficient set. Such scaling only increases the predictive ability of the network by replicating the underlying convolutional operations and structure of the network. EfficientNet uses the MBConv blocks as in the MobileNet V2 network, but with a squeeze-and-excitation (SE—[50]) block being added. This structure helps reduce the overall number of operations required and the model’s size.

The backbone CNN extracts from the image features that uniquely describe different locations and builds embedding vectors that serve as global image descriptors in our system. In the next step, the algorithm creates an index from the global map, which is used for efficient similarity search. The original images collected by the robot are no longer needed for localization and the obtained global map has a compact form. All operations to produce the global map are performed offline.

Then, to localize the robot, we need to query the global map (database of embeddings) with the descriptor/embedding produced from the current perception of the agent, which boils down to a similarity search task. Similarity search is a typical issue in machine learning solutions using embedding vectors, and becomes increasingly difficult as the vectors’ dimensions and/or size increase. Classic methods for finding similarity between vector-described elements in an extensive database include linear search and search in K-D-trees [51]. K-D-trees are binary trees used to organize points representing data in a K-dimensional space and allow for a very efficient search of points in that space, including a nearest neighbour (NN) search, which we are interested in [52].

Each node in the tree represents a K-dimensional point. Each nonleaf node in the tree acts as a hyperplane, dividing the space into two parts. Using a K-D tree for nearest neighbour search involves finding the point in the tree that is closest to a given query point. For this purpose, the algorithm traverses the tree and compares the distance between the query point and points in each leaf node. Starting from the root node, it recursively moves down the tree until it reaches the leaf node, following the same procedure as when inserting a node. Many implementations of the nearest neighbour search using K-D-trees are known in Python, including the very popular SciKit-Learn library. However, for this project, we selected the Facebook AI Similarity Search (Faiss) library [53], written in C++ with wrappers for Python and support for GPU, which suits our implementation on Nvidia Jetson well. The Faiss library solves our similarity search problem using indexing and searching with the KNN method. Once the index type is selected, the algorithm processes the embedding vectors obtained from the neural network and places them in the index. The index can be stored on disk or in memory, and searching, adding, or removing items to the index can be performed in real-time. In addition, the Faiss library has an autotuning mechanism that scans the parameter space and selects those parameters that provide the best possible search time at a given accuracy.

Place recognition begins by loading the learned CNN model and index of images (map) into memory, and then the captured images (queries) are compared with the previously created image database using the KNN algorithm in the space of embedding vectors. The embeddings are compared using L2 (Euclidean) distance, which has been shown to be more computationally efficient than feature binarization followed by the comparison applying Hamming distance [36,54].

Once the similarity between the query image and the map is determined, the results are presented in the form of the image retrieval accuracy and the position error between the query image and the map image determined as the most similar one. As we assume that ground truth positions for all map images and query images are known, as in the COLD dataset [55], we simply use the Euclidean distance in metric space to quantify this error. The averaged Euclidean distance is used to calculate the position error over an entire experiment involving many queries. The arithmetic mean is calculated over all places according to the equation:

(2) $$\bar{\bar{b_{L}}}\;=\;\frac{\sum_{i=1}^{n}\sqrt{{\left(x_{{\mathrm{gt}}_{i}}-x_{e_{i}}\right)}^{2}+{\left(y_{{\mathrm{gt}}_{i}}-y_{e_{i}}\right)}^{2}}}{n},$$

where $\bar{\bar{b_{L}}}$—the average position measurement error; n—the number of query images; $x_{gt_{i}}$—the x coordinate for the ground truth location of the *i*-th query image; $x_{e_{i}}$—the x coordinate for the estimated location of the *i*-th query image; $y_{gt_{i}}$—the y coordinate of the estimated location of the *i*-th query image; $y_{e_{i}}$—the y coordinate for the estimated location of the *i*-th query image.

The architecture of the localization system shown in its general form in Figure 1 was tested in several variants differing in the type of neural network used as an extractor of image embeddings and the use of catadioptric camera images directly or images converted to panoramic form. The suitability of the NetVLAD approach in the described system was also investigated. The investigated variants are described in the next section of this paper.

## 4. Experiments

To confirm the proposed solution’s effectiveness and determine the best-performing CNN architecture, experiments were carried out at the Mechatronics Centre of Poznań University of Technology using two catadioptric cameras with different parameters. Then, experiments with the publicly available COLD database were carried out to demonstrate the performance of our approach with respect to selected state-of-the-art solutions in appearance-based localization on this dataset.

### 4.1. Experiment 1: Integrated Sensor on a Mobile Robot

**Figure 3:**
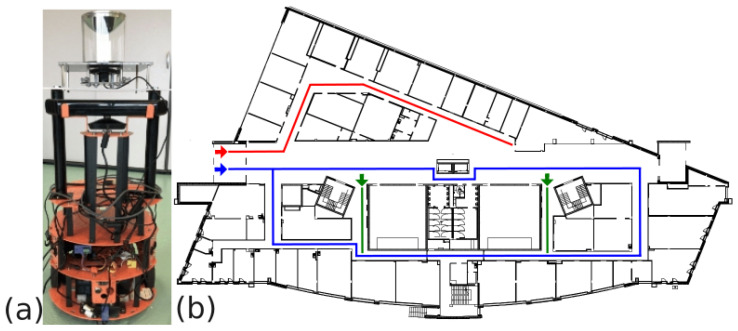
Labbot mobile robot with the integrated sensor with a catadioptric camera (**a**); robot paths during image collection—different colours indicate different paths (**b**).

A Labbot robot (Figure 3a) with an integrated catadioptric vision sensor was used in the first scenario. The catadioptric camera in this sensor consists of a Microsoft LifeCam and a hyperbolic mirror, which provides a field of view of 360${}^{\circ }$ and produces images with a resolution of 640 × 480. The images are processed by a Nvidia Jetson TX2 computer integrated with the sensor [8] The Jetson TX2 offers a 256-core Pascal architecture General Purpose Graphics Processing Unit (GPGPU) to support the real-time operation of the localization system.

**Figure 4:**
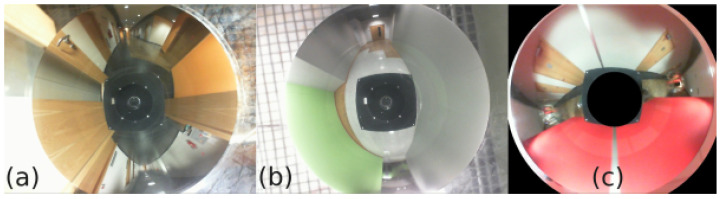
Omnidirectional images of different locations (**a**,**b**) in the Mechatronics Centre and an example image after masking (**c**).

The considered dataset contains 606 images (Figure 4a,b), which were recorded on three floors of the Poznań University of Technology Mechatronics Centre building (Figure 3b). All images were subjected to a masking process (Figure 4c) to remove areas that did not contain useful information. Using the localization system described in Section 3, embeddings of 2048 × 1 in size were calculated for each image and registered in a database of 2048 ×*n* in size, which is a global map based on *n* reference images (*n* = 484 in the experiment). The robot’s main localization task uses the integrated sensor’s Jetson platform in real time. The configuration of the localization system in this experiment was the following:

- Raw catadioptric images were used (cf. Figure 4) without converting them to panoramic images.
- The neural network used to produce the embeddings was EfficientNet, which was selected upon literature-based analysis.

The EfficientNet architecture has gained prominence as an effective solution for image processing on edge devices due to its remarkable balance between accuracy and efficiency. By leveraging techniques like compound scaling, which uniformly scales the network width, depth, and resolution, EfficientNet optimizes the model’s architecture to maximize accuracy while minimizing the number of parameters and computations. This enables real-time inference and efficient utilization of resources on edge devices, ensuring faster and more responsive image processing capabilities even with limited computing power [56]. Moreover, in the considered application, the input size of the available pretrained EfficientNet B5 models matches the resolution of our target images.

The used EfficientNet B5 has 577 layers and the input image size is (456,456,3). This network has high accuracy with a relatively small number of model parameters, which positively affects the processing speed of the embedded system. The network was fine-tuned before use because EfficientNet B5 was pretrained on images from the ImageNet dataset. This process was implemented using a dataset of about 10,000 augmented omnidirectional images produced from the previously collected database of 606 original images. Only standard augmentation methods available in the TensorFlow environment were applied to the images.

A practical problem in the scenario considered in Experiment 1 was the high similarity of the indoor environment in the Mechatronics Centre building. Images were obtained approximately every 0.5 m along the robot’s path, and adjacent images in the database are very similar and often indistinguishable, even by a human. Therefore, the entire dataset was manually divided into 17 different sections, each describing a topologically different location (represented by different colours in Figure 3b). Due to this organization of the dataset, no ground truth positions are provided for particular images, and we can assess the localization results only in terms of the image retrieval accuracy for particular sections. The localization process is then performed only for these 17 locations, with each location represented by 30 to 40 acquired images that partially overlap. In the training process, each section was divided into training sequences (60%), validation sequences (20%), and test sequences (20%).

### 4.2. Experiment 2: Stand-Alone Catadioptric Camera

The good results obtained in the preliminary experiment with the mobile robot motivated us to extend this research with a catadioptric camera of a different mechanical design and better parameters, as the relatively small horizontal field of view and often blurred images were the main drawbacks in the previous experiment.

The field of view of a catadioptric camera depends on the shape and size of the mirror being used [57]. A catadioptric sensor captures a wider field of view by using lenses and mirrors that need to be arranged carefully. Designing the mirrors is crucial to ensure a single effective viewpoint, which is necessary for generating pure perspective images from the sensed images [58]. In the new experiment, the integrated sensor was replaced by a catadioptric vision sensor consisting of a professional Basler acA2440-35uc camera with a Kowa 4.4–11 mm lens [59] and a hyperbolic mirror, whose field of view is much larger than the mirror used in the previous experiment. A hyperbolic mirror allows us to obtain the single effective viewpoint of the camera–mirror system using typical camera lenses [60], while the mirror we use in this design is larger than the previous one, and is attached at a larger distance from the camera. Both these factors contribute to a much larger horizontal field of view.

**Figure 5:**
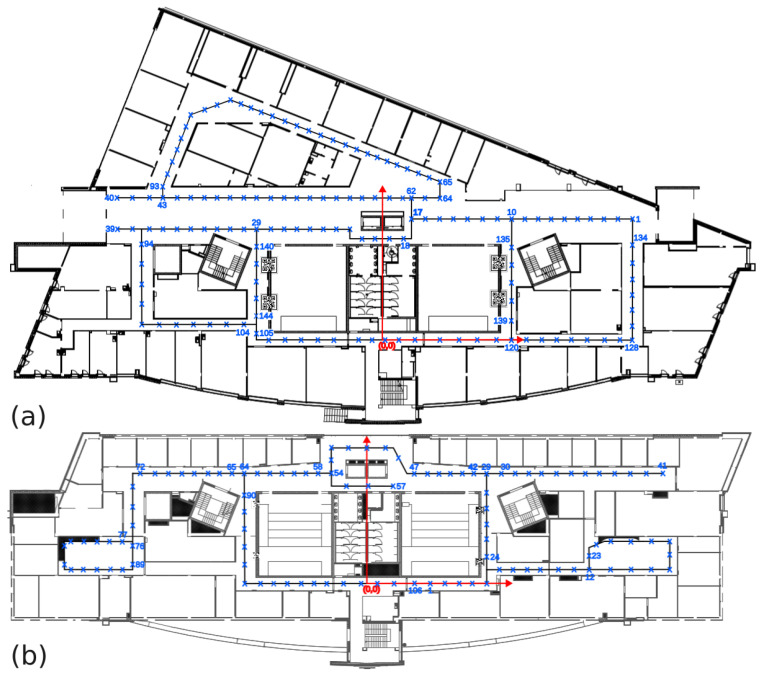
Blueprint of the first floor (**a**) and third floor (**b**) of the Mechatronics Centre building, with marked places (blue crosses) where images were taken.

**Figure 6:**
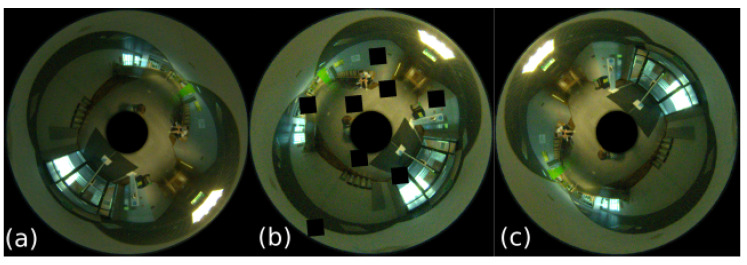
Example of omnidirectional image augmentation: (**a**)—original picture; (**b**,**c**)—augmented images.

Images with a resolution of 1080 × 1440 were taken for two floors of the same Mechatronics Centre building: the first floor was divided into 144 places (Figure 5a) and the 3rd floor into 106 places (Figure 5b). Ground truth positions of the acquired images were obtained by measuring the position of the sensor manually with tape with respect to the known floor plan of the Mechatronics Centre building. Due to the augmentation process (Figure 6), the collection of images for training purposes increased to about thirty thousand images (details are given in Table 1).

In this experiment, the collected set of images was used to compare the quantitative results of place recognition for panoramic and omnidirectional images for three different CNN architectures: EfficientNet B7 [61], EfficientNet V2L [62], and MobileNetV2 [48]. Moreover, to investigate different strategies for creating the reference database (global map), the dataset was tested in three different configurations:

- Configuration A—the entire dataset was divided into a training set (60%), a validation set (20%), and a test set (20%) for each place. The validation set was then used as the reference database of embeddings.
- Configuration B—the entire dataset was divided into a training set (60%), a validation set (20%), and a test set (20%) in such a way that the locations next to the places represented in the test set were always represented in the map of embeddings. The global map of embeddings was created from a combination of the training and the validation set, but the places from the test set, used then as queries, were not directly represented in the map.
- Configuration C—all images of the places located on the first floor were divided into a training set (80%) and a validation set (20%). The set of images recorded on the third floor was used to test the proposed solution. The 106 places for which images were recorded on the third floor were divided into the database of embeddings (80%) and a test set used as queries (20%), in such a way that the locations next to the places included in the test set were represented in the map of embeddings.

For each network configuration and dataset, the training process was conducted as in Experiment 1, with the pretrained backbone network, and by fine-training the last layers of this network on the target training dataset constructed according to the concept defined above for the given configuration.

As a follow-up of this experiment, we tested with the same dataset the NetVLAD architecture for comparison with our approach. It was shown in [35] that the NetVLAD architecture achieves the best-placed recognition results with the AlexNet and VGG-16 used as backbone networks. Hence, in order to compare our approach to place recognition, which is relatively simple, to the state-of-the-art NetVLAD architecture, we used a Python language implementation of NetVLAD [63] with the VGG-16 backbone. The NetVLAD model was subject to the same training process as in the case of our system, with the training sets defined in Configurations A, B, and C.

### 4.3. Experiment 3: COLD Datasets

An important related work to our research is the article by Cabrera Mora et al. [43], which presents several different configurations of the AlexNet network producing embeddings used for appearance-based localization with omnidirectional images without panoramic conversion. The task of the trained neural network is to perform rough localization (room identification) and then metric localization for the identified room by searching for the place closest to the query embedding. The experiments presented in [43] used images available in the Freiburg dataset, which is part of the publicly available COsy Localization Database [55]). This inspired us to replicate some of the experiments from [43] using our approach to localization. Using the same dataset and experiment design gives a chance for a fair comparison of quantitative results, which is usually not available in the not-so-common research on localization with omnidirectional images.

**Figure 7:**
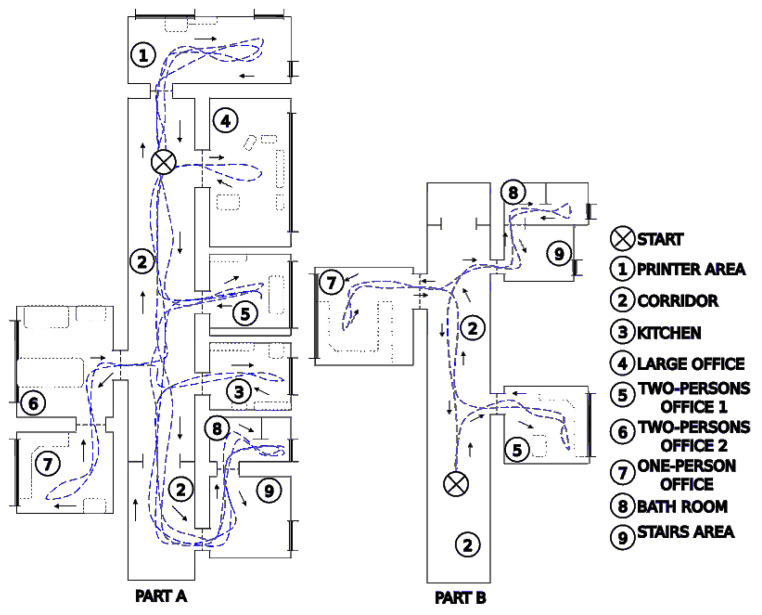
Maps of the two parts of the laboratory in Freiburg with approximate paths followed by the robot during data acquisition (map and trajectories data adopted from the COLD dataset web page https://www.cas.kth.se/COLD/cold-freiburg.html).

Moreover, we consider COLD Freiburg an interesting dataset on its own, as it contains omnidirectional images captured by a robot that followed a number of different paths in a building at the University of Freiburg. The robot visited various rooms, such as the kitchen, corridors, printer areas, bathroom, and offices (Figure 7). These rooms have wide windows and glass walls, making visual localization a particularly challenging task. The collection of images was collected under real conditions, e.g., changes in furniture, people being on the move, changes in lighting conditions (cloudy days, sunny days and nights), etc. Moreover, the images were captured while the robot was moving; therefore, they may contain blurring effects or other dynamic changes. What is important is that accurate ground truth positions of the captured images are provided in this dataset thanks to the laser scanner localization of the robot. The ground truth positions were used exclusively to measure the metric localization errors.

**Figure 8:**
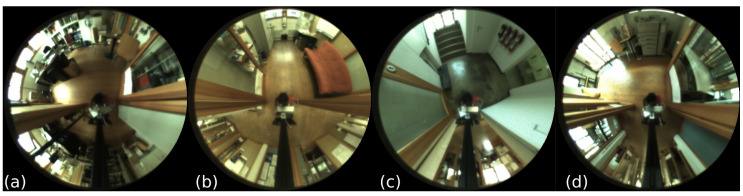
Example images from the COLD Freiburg dataset: (**a**) one-person office (1PO-A); (**b**) kitchen (KT-A); (**c**) stairs area (ST-A); (**d**) printer area (PA-A).

In order to evaluate the influence of the changing lighting conditions on the localization task, it was proposed in [43] to use as training data only images recorded on cloudy days, whose acquisition locations are about 20 cm apart. On the other hand, in order to assess the robustness of the location to changes in illumination, images captured on sunny and cloudy days and at night were used for testing. The COLD Freiburg dataset contains images captured in nine different rooms: a kitchen, a bathroom, a printer area, a stairwell, a long corridor, and four offices (Figure 8).

In order to evaluate the appearance-based localization system proposed in this paper, a direct comparison was made with the solution presented in [43]. To facilitate a fair comparison, we made an attempt to replicate the sets of images used in the experiments described in [43]. However, starting from the same images of the Freiburg dataset, we used only our own processing pipeline; in particular, each training set was augmented by darkening random portions of the images, rotating them, and changing the illumination. In our case, the training set was also the global map of embeddings.

Training dataset number one, which is an exact replication of the dataset from the work of [43], was obtained from a set of images taken during a cloudy day, and it was downsampled to obtain a set of images describing locations at an average distance of 20 cm between the acquisition points of successive images along the robot’s path. Detailed information on the number of images contained in the training set depending on the type of lighting conditions and room is provided in Table 2 and Table 3. Verification of the correctness of the obtained model for embedding generation was carried out for three test sets of query images: the first set consists of images captured on cloudy days but not included in the training set (2595 images); the second test set contains all images captured on sunny days (2807 images); and the third test set consists of all images captured at night (2876 images).

## 5. Results and Discussion

This section presents and discusses the results of the three experiments described in this paper. Quantitative results in terms of place recognition (i.e., image retrieval) accuracy are presented for all experiments. For experiments no. 2 and no. 3, we also present quantitative results in terms of the metric localization accuracy, as the datasets used in these experiments provide ground truth for positions of the place images in a global reference system. Moreover, we discuss qualitative localization results, pointing out the most common sources of localization errors and providing recommendations for training strategies of the deep neural networks that make the resulting models robust to changes in the environment.

### 5.1. Experiment 1

**Figure 9:**
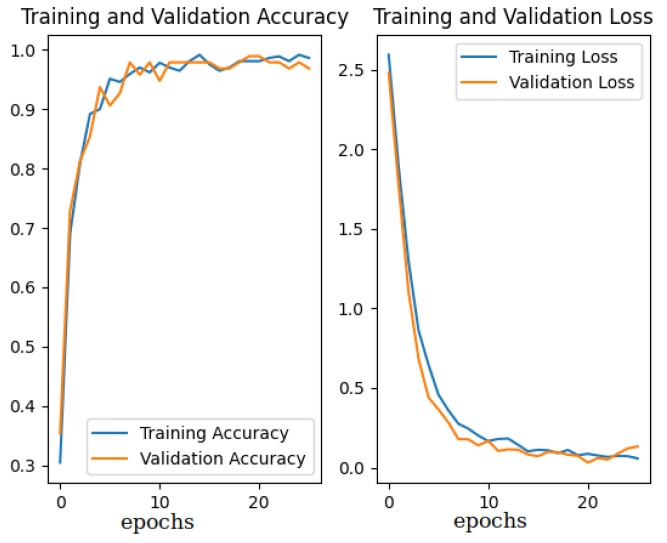
Model training results in Experiment 1.

As defined in Section 4, in Experiment 1, appearance-based localization was conducted for 17 sections, each of them containing several image acquisition locations, and being a description of a larger corridor space. The best network training results were obtained for the unfrozen last 50 layers of the backbone CNN, a learning rate of $1\times 10^{-4}$, and a batch size of 16, with a learning error of 0.1605, learning accuracy of 0.9596, validation error of 0.1183, and validation accuracy of 0.9796 (Figure 9).

**Figure 10:**
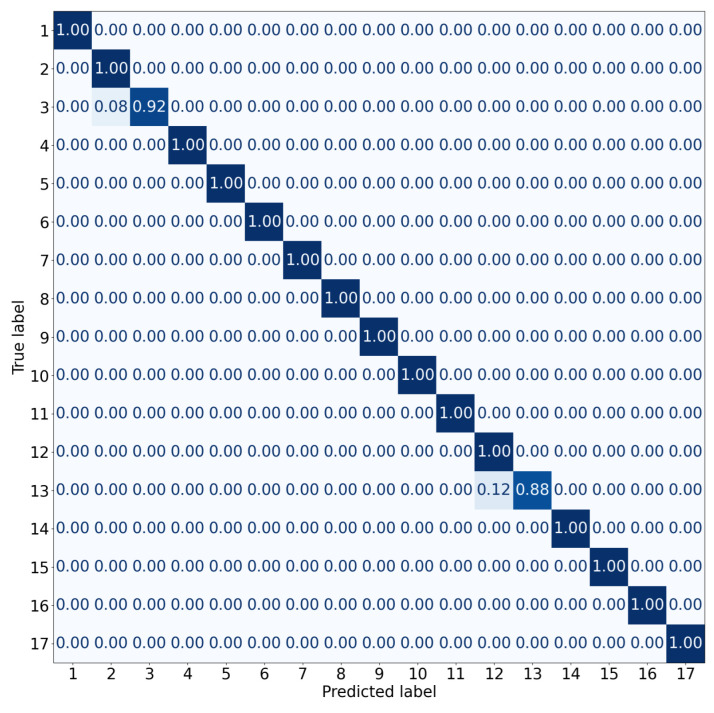
Confusion matrix for 17 sections.

**Figure 11:**
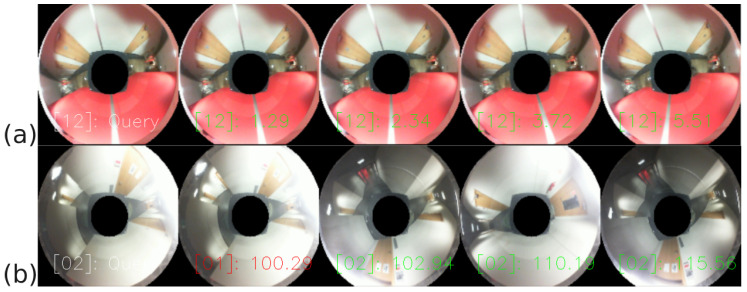
Results of sample section predictions. The image in the first column is a query; the other columns are the four closest neighbours. In square brackets, there is the section number (i.e. [12], [02]), and next to it, the L2 distances between the query and the presented image are given. An example of (**a**) correct place recognition and (**b**) mismatched sections having slightly overlapping ranges.

On the test dataset containing 122 query images, the average accuracy of place recognition was 98%, with very few misclassified queries, as shown by the confusion matrix in Figure 10. The average processing time of a single query image was 480 ms, with a standard deviation of 83ms and a maximum time of 1313 ms, allowing for real-time localization. A qualitative example of place recognition is given in Figure 11. Visual inspection of the results and the confusion matrix suggest that the most common section mismatch is when the same place is at the start of a new section and the end of a previous section. However, errors also are caused by blurred images and bright spots of sunlight or artificial light in the images.

This experiment allowed us to conclude that the proposed approach to appearance-based localization with embeddings produced by a lightweight CNN suits the target application in terms of both image retrieval accuracy and real-time performance. However, the used sensor, having rather a small field of view and mechanical structure prone to decalibration and defocusing (causing blurred images), did not allow us to extend these investigations to a larger dataset with ground truth positions of images.

### 5.2. Experiment 2

The conclusions drawn from Experiment 1 were taken into account while designing the next experiment involving the use of a different catadioptric sensor and a more extended and diversified dataset of indoor images with ground truth positions.

**Figure 12:**
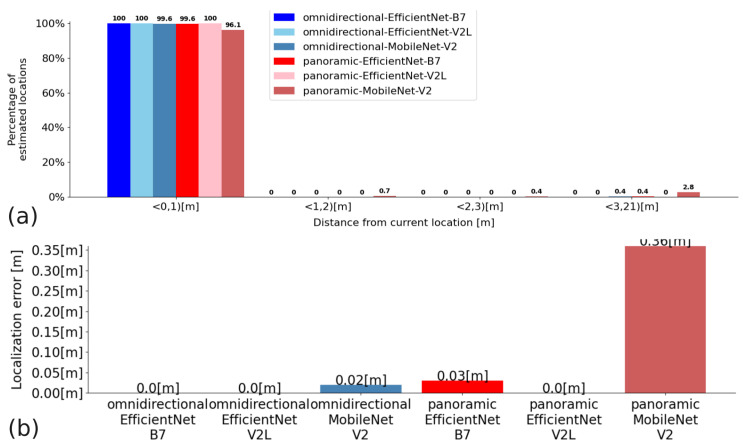
Quantitative results for Configuration A: (**a**)—a percentage of matches that are within a range of distance from the actual distance (the units on the x-axis are the ranges of distances); (**b**)—average distance measurement error.

**Figure 13:**
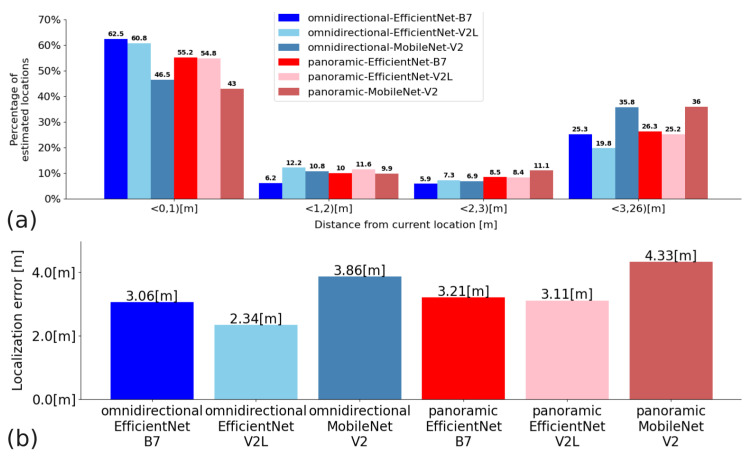
Quantitative results for Configuration B. (**a**)—the percentage of matches that are within a range of distance from the actual distance (the units on the x-axis are the ranges of distances); (**b**)—the average distance measurement error.

**Figure 14:**
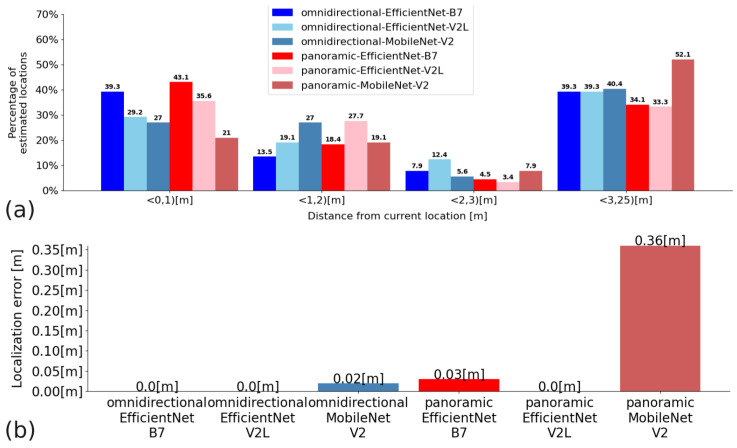
Quantitative results for Configuration C: (**a**)—the percentage of matches that are within a range of distance from the actual distance (the units on the x-axis are the ranges of distances); (**b**)—the average distance measurement error.

In Experiment 2, the convolutional neural networks EfficientNet B7, EfficientNet V2L, and MobileNet V2 were compared for Configurations A, B, and C. The quantitative results presented in the graphs show the percentage of Cartesian locations found in the given distance intervals expressed in meters, and the average Euclidean distance measurement error: for Configuration A—Figure 12; Configuration B—Figure 13; and Configuration C—Figure 14. Moreover, Table 4 shows the average time of processing a single query image with the proposed solution based on embeddings and Faiss KNN search.

A mismatch of the neighbouring places occurs only when the images overlap significantly (note that the catadioptric camera used in Experiment 2 has a much larger field of view that the previously used one) and are very similar to each other due to the self-similar nature of the environment. No significant difference was noticed between the results obtained for omnidirectional and panoramic images, which indicates that for appearance-based localization with our approach, it is unnecessary to convert the omnidirectional images to panoramic images. Hence, we can avoid the time-consuming conversion and rectification procedure [8], without compromising the results.

As a follow-up of Experiment 2, a comparison of localization results was performed between the NetVLAD approach with VGG-16 and VLAD layer, and our approach with two variants of the EfficientNet backbone. This comparison was performed for all three configurations of the reference map (A, B, and C) and both the omnidirectional and converted panoramic images.

Quantitative results of the average Euclidean distance measurement error for all configurations considered in this test are shown in Table 5. As can be seen in this table, for the panoramic images, the proposed solution has a smaller average error for distance measurement than NetVLAD. On the other hand, for original omnidirectional images, the proposed solution with EfficientNet and embedding has the same or larger average error for distance measurement than NetVLAD with VGG-16.

From Experiment 2, we conclude that the CNN architecture performing best in our system is EfficientNet V2L, a model from a recently introduced family of convolutional networks that achieve faster training and better parameter efficiency than older network models [62]. This model, being up to 6.8 times smaller than state-of-the-art models, suits our embedded computing platform well. Moreover, our approach performs as a pair with the much more complicated and much bigger NetVLAD architecture. On the other hand, Experiment 2 shows that NetVLAD can handle raw omnidirectional images without converting them to panoramic images if it is trained on a representative dataset. As to the strategy of defining the reference map and the training dataset, the results of Experiment 2 show that it is possible to find a correct neighbouring place, even if the very exact image of the queried place is not included in the reference map. However, these results also show that the generalization ability of the investigated deep learning solutions, including NetVLAD, is somewhat limited if the query images come from a different environment than the training set. This is suggested by the worse results in Configuration C, no matter if omnidirectional or panoramic images are being used. Therefore, the generalization ability of the proposed architecture should be further investigated.

### 5.3. Experiment 3

Conclusions about the limited generalization ability of the proposed localization system drawn from Experiment 2 were one of the main motivations behind the concept of Experiment 3, which applies the publicly available COLD Freiburg dataset and compares side-by-side to the results obtained on the same dataset and published recently in [43].

In [43], the best results were obtained for the retrained AlexNet, with 97.11% of correct room identifications obtained for images captured during cloudy daytime, 93.48% for sunny days, and 96.77% for nighttime.

**Figure 15:**
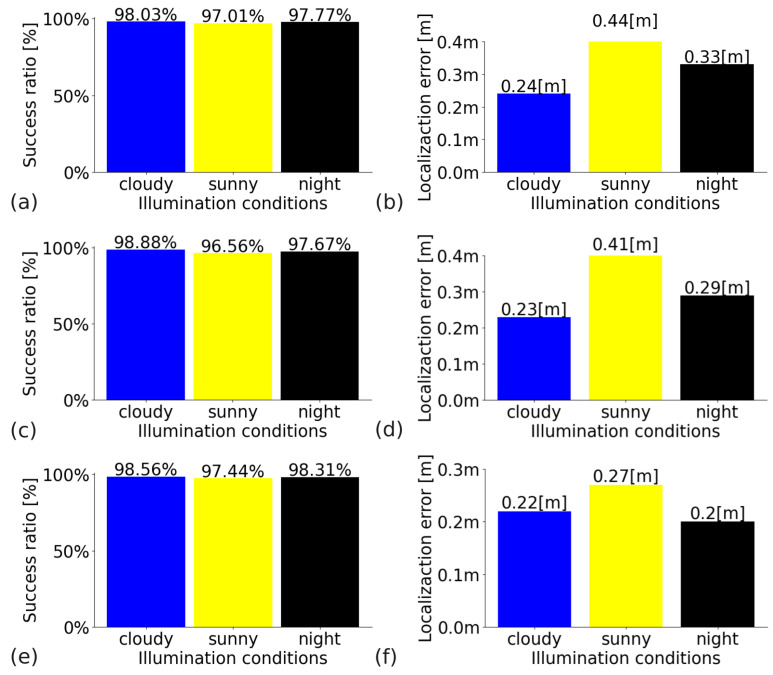
Success ratio for EfficientNetV2L and the set of embeddings acquired from the training set for the room search task for the COLD Freiburg dataset. The result obtained under cloudy (blue), night (black), and sunny (yellow) conditions for the model learned on the training set, namely, a set of images on cloudy days (**a**), a set of images on cloudy days extended by missing acquisition locations found in images for sunny days and night (**b**), and a balanced set of images obtained on cloudy and sunny days and at night (**c**). Average location error in meters for a set of images on cloudy days (**d**), a set of images on cloudy days extended by missing acquisition locations found in images for sunny days and at night (**e**), and a balanced set of images obtained on cloudy and sunny days and at night (**f**).

Following the methodology of replicating the selected experiments from [43] with respect to the used images, we achieved the following results for appearance-based localization using embeddings with EfficientNet V2L: 98.03% for cloudy days, 97.01% for sunny days, and 97.77% for nights. The full quantitative results are presented in Figure 15. Thus, better accuracy of room recognition was obtained for cloudy days by 0.92%, by 3.53% for sunny days, and by 1% for images recorded at night. The average distance measurement error for the room was also analysed, and the results obtained are presented in Figure 15b. The proposed solution achieved a smaller average distance measurement error than in [43] for cloudy days by 0.05 m, and by 0.24 m for sunny days; only for images taken at night was the average error increased by 0.04 m.

During the analysis of the results, it was noticed that the images recorded on sunny days and nights have different acquisition spots (positions) than those acquired for cloudy days, and some of them are farther than 20 cm from the acquisition spots for cloudy days, e.g., for the room labelled CR-A, the farthest acquisition spot on sunny days was 0.5 m away from the farthest acquisition spot on a cloudy day. For this reason, an extension of the training set no. 1 was made, only with images taken at the missing acquisition spots for sunny days and for nighttime (Table 2 and Table 3). Note that the images added to the learning set were removed from the test sets, and never used as queries. The results obtained with this amendment of the training set are shown in Figure 15c,d. However, neither in the case of room identification accuracy nor for position determination errors did the results improve significantly.

Based on the data from Table 2, it was noticed that the training dataset number 2 is not balanced in terms of images of a given room depending on lighting conditions. In deep learning, several techniques are commonly used to handle imbalanced datasets of images. One approach is oversampling, where the minority class samples are replicated to match the majority class. Another technique is undersampling, where random samples from the majority class are removed to balance the dataset. Additionally, there are methods like synthetic data generation, cost-sensitive learning, and ensemble techniques [64]. However, these methods have certain drawbacks. Oversampling can lead to overfitting and a loss of generalization ability. Undersampling can discard valuable information and result in underrepresented classes being ignored. Synthetic data generation may introduce unrealistic patterns. Cost-sensitive learning requires careful tuning of class weights. There is no technique that universally addresses all imbalanced dataset challenges. For this reason, a third training set was created (no. 3), which contains images representing acquisition locations about 20 cm apart for each room at night and on sunny and cloudy days (Table 2 and Table 3). Finally, for this illumination-balanced training set, the best results were obtained for room identification: 98.56% for cloudy days, 97.44% for sunny days, and 98.31% for nights (Figure 15e). Also, the smallest average distance measurement error was obtained for training with the set no. 3: 0.22 m for cloudy days, 0.27 m for sunny days, and 0.2 m for nights (Figure 15f). Based on the data from Table 3, it can be seen that the percentages of the images of each room in each training set are very close to each other, but only by balancing the set by the lighting conditions (i.e., sunny, cloudy, night) were satisfactory results obtained.

**Figure 16:**
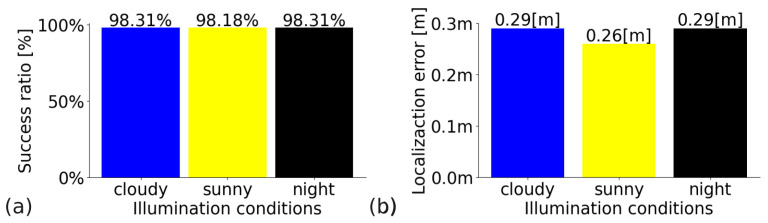
Success ratio for EfficientNetV2L and the set of embeddings acquired from the training set for the room search task for COLD Saarbrücken dataset for part B. Results obtained under cloudy (blue), night (black), and sunny (yellow) conditions for the model learned on the training set are a balanced set of images obtained on cloudy and sunny days and night (**a**). Average location error in meters for a balanced set of images obtained on cloudy and sunny days and night (**b**).

In order to explore the generalizability of the proposed system to other environments, we also demonstrate localization results for the Saarbrücken sequence from the COLD dataset. This is a sequence of images collected in a different location within a building having different characteristics than the one used for the Freiburg sequence [20]. Both Freiburg and Saarbrücken sequences from the COLD dataset were used in the research on mobile robot’s localization presented in [32], although the inaccurate description of the dataset used in this paper does not allow us to replicate the Saarbrücken sequence experiment for direct comparison, as it was accomplished for the Freiburg sequence used in [43]. Therefore, considering the analysis of the influence of an imbalanced training dataset on the localization accuracy of the system, it was decided to use only part B of the Saarbrücken sequence, since it is the only one with images for all weather and lighting conditions. The results presented in Figure 16 confirm that our approach generalizes well to different indoor environments, achieving a similar success rate of image retrieval and a similar average localization accuracy as the Freiburg sequence.

The hierarchical localization of the mobile robot in [43] is divided into two subtasks: the first one is room recognition, and the second one is accurate localization within the known room. Accurate localization involves estimating the position in which the test image was captured using a nearest neighbour search method in the space of global descriptors obtained from the convolutional network, which is similar to our approach. In the paper, several experiments with the COLD dataset were performed based on different configurations of the networks and training sets used in the room classification task. Finally, the authors of [43] selected the CNN network trained in experiment number 2 and the fully connected layer numbered 6 in their network as the one producing the global descriptors from images, because this configuration showed the greatest robustness to changes in lighting conditions in the preliminary experiments. This configuration is referred to as CNN2 + FC6. Another configuration considered in [43] is the CNN network trained in experiment 8 and the output of the sixth layer (CNN8 + FC6).

Table 6 presents the quantitative results and computation times obtained for our appearance-based localization. The average processing time per image was 0.32 seconds (achieving 3.13 FPS), making appearance-based localization in real-time in the context of the typical motion speed of our service robot. For comparison purposes, this table also contains results for the two selected configurations of the system from [43], and results of other hierarchical localization methods (not learning-based) investigated in [32] and used for comparison in [43]. Note that the numerical results from [32,43] are taken from the respective papers, as we did not attempt to reimplement these systems, replicating only the training and test sets for Experiment 3.

The appearance-based localization methods studied in [32] include the global HOG and Gist descriptors. HOG is a feature decoder often used in image processing for object detection. The Gist descriptor, on the other hand, is used to extract global features of the environment by combining visual and semantic information through a set of perceptual dimensions that represent the dominant spatial structure of the scene. Both HOG and GIST are descriptors that do not use machine learning algorithms, providing an interesting reference point for the method proposed in the article.

The results shown in Figure 15 and in Table 6 document that the approach to appearance-based localization proposed in this paper, despite being simple and lightweight, outperforms some recently published results in this area. Considering the fact that our solution turned out to be similar in performance to the state-of-the-art NetVLAD deep learning architecture, which requires many more computing resources, we conclude that our research reached its goal, demonstrating a versatile and accurate deep learning architecture that is suitable for the low-cost Nvidia Jetson TX2 computing platform. However, the main scientific outcome of Experiment 3 seems to be demonstrating how important it is to prepare a balanced dataset for training, particularly in the context of generalization over various image acquisition characteristics.

## 6. Conclusions

The results of the tests of the place recognition software for catadioptric cameras and the edge computing platform allow us to conclude that the proposed neural network architecture and parallel processing make it possible to obtain a real-time localization system that works with raw catadioptric images, despite their distorted nature.

The extensive study of the algorithm of appearance-based localization and comparison of results with similar solutions known from the literature demonstrate that the proposed approach makes it possible to obtain highly descriptive embeddings of the observed locations, and consequently, efficient appearance-based localization.

The most important conclusions, summarizing the remarks discussed in Section 5, concern the best performance of the EfficientNet V2L CNN backbone for generating the embeddings and the pivotal importance of preparing a well-balanced training set for this network, even if transfer learning with pretraining on a large dataset of general purpose images is used. A practical conclusion is that the not-so-recent and low-cost Nvidia Jetson TX2 embedded computer is enough to run a carefully engineered deep learning system for appearance-based localization. This opens interesting opportunities for developing affordable service and social indoor mobile robots utilizing a catadioptric camera as the main localization sensor.

However, a limitation of the proposed appearance-only approach to global localization is the limited accuracy of the obtained metric position of the robot. This accuracy depends on the density of the global map, because the obtained position of the robot is defined by the known location of the most similar image. If the images were collected close to each other, then the position of the robot can be determined more accurately, but if the distances between the points where the images were captured are large, the accuracy is decreased. This limitation will be addressed in our further research by implementing a neural network that will regress the position of the robot with respect to the reference image retrieved from the map. Further research on this system will also concern the implementation of triplet loss with hard negative mining, as this training scheme turned out to be very effective in a number of localization systems. This training strategy should allow the network to develop more specific features, thus making the localization system more effective in highly repetitive indoor environments.

## Figures and Tables

**Table 1:** Number of images in training and validation datasets for Configurations A, B, and C. Numbers in brackets denote the number of images after augmentation.

	Configuration A		Configuration B		Configuration C	
	Training	Validation	Training	Validation	Training	Validation
	Dataset	Dataset	Dataset	Dataset	Dataset	Dataset
omnidirectional	994 (25,844)	250 (6500)	959 (24,934)	241 (6266)	753 (19,578)	288 (7488)
panoramic	2982 (77,532)	750 (19,500)	2611 (67,886)	653 (16,978)	2259 (58,734)	864 (22,464)

**Table 2:** The number of images of each room depending on the weather for three training sets, where n.i. is the number of images and % is the percentage of images of a particular room depending on the weather conditions. Dataset 1—a set of images recorded only on cloudy days. Dataset 2—set 1 extended by the missing acquisition points found in the sets for sunny days and nights. Dataset 3—a set of images showing acquisition points located every 20 cm for images recorded in all types of weather conditions.

	Training Dataset 1		Training Dataset 2						Training Dataset 3					
	(575 Images)		(820 Images)						(1801 Images)					
	Cloudy		Cloudy		Sunny		Night		Cloudy		Sunny		Night	
	n.i.	%	n.i.	%	n.i.	%	n.i.	%	n.i.	%	n.i.	%	n.i.	%
Room	575	100	576	70.2	139	17.0	105	12.8	573	31.8	651	36.2	577	32.0
1PO-A	45	100	47	69.1	15	22.0	6	8.8	46	31.3	54	36.7	47	33.0
2PO1-A	52	100	50	79.4	8	12.7	5	8.0	48	36.9	47	36.3	35	26.9
2PO2-A	33	100	30	58.8	8	15.7	13	25.5	34	30.4	40	35.7	38	33.9
CR-A	248	100	249	76.9	43	13.3	32	9.9	247	33.2	267	35.9	229	30.8
KT-A	43	100	41	42.3	31	32.0	25	25.8	40	19.9	79	39.3	82	40.8
LO-A	32	100	31	62.0	12	24.0	7	14.0	34	33.7	35	34.7	32	31.7
PA-A	58	100	58	82.9	8	11.4	4	5.7	58	37.2	55	35.3	43	27.7
ST-A	31	100	33	76.7	5	11.6	5	11.6	31	31.3	36	36.4	32	32.3
TL-A	33	100	37	68.5	9	16.7	8	14.8	35	31.3	38	33.9	39	34.9

**Table 3:** The number of images of each room depending on its type in a given training set, where n.i. is the number of images and % is the percentage of images of a given room depending on weather conditions. Training dataset 1—a set of images recorded only on cloudy days. Training dataset 2—dataset 1 extended by the missing acquisition points found in the datasets for sunny days and nights. Training dataset 3—a set of images showing acquisition points located every 20 cm for images recorded in all types of weather conditions.

	Training Dataset 1		Training Dataset 2		Training Dataset 3	
	(575 Images)		(820 Images)		(1801 Images)	
Room.	n.i.	%	n.i.	%	n.i.	%
1PO-A	45	7.83	68	8.29	147	8.16
2PO1-A	52	9.04	63	7.68	130	7.22
2PO2-A	33	5.74	51	6.21	112	6.22
CR-A	248	43.13	324	39.51	743	41.25
KT-A	43	7.48	97	11.83	201	11.16
LO-A	32	5.57	50	6.1	101	5.61
PA-A	58	10.09	70	8.54	156	8.66
ST-A	31	5.39	43	5.24	99	5.50
TL-A	33	5.74	54	6.59	112	6.22

**Table 4:** Mean Euclidean distance error ($\bar{\bar{b_{L}}}$) and mean time ($\bar{\bar{t}}$) of location determination on the Jetson TX2 computing platform for original (omnidirectional) and panoramic images.

Experiment 2										
**Neural**	**Image**	**Configuration A**			**Configuration B**			**Configuration C**		
**Network**	**Type**	$\bar{\bar{b_{L}}}$ **[m]**	$\bar{\bar{t}}$ **[s]**	${\bar{\bar{t}}}_{\mathrm{tr}}$ **[h]**	$\bar{\bar{b_{L}}}$ **[m]**	$\bar{\bar{t}}$ **[s]**	$t_{\mathrm{tr}}$ **[h]**	$\bar{\bar{b_{L}}}$ **[m]**	$\bar{\bar{t}}$ **[s]**	$t_{\mathrm{tr}}$ **[h]**
EfficientNet B7	omni	0.00	0.52	2.15	3.06	0.48	3.25	4.43	0.47	2.16
EfficientNet B7	panoramic	0.03	0.56	37.21	3.21	0.49	16.24	3.92	0.50	11.30
EfficientNet V2L	omni	0.00	0.35	1.98	2.34	0.35	3.84	4.94	0.34	2.07
EfficientNet V2L	panoramic	0.00	0.39	14.54	3.11	0.37	15.46	3.60	0.36	12.14
MobileNet V2	omni	0.02	0.08	2.24	3.86	0.07	3.15	5.01	0.07	1.55
MobileNet V2	panoramic	0.36	0.11	16.32	4.33	0.11	15.56	6.87	0.11	11.53

**Table 5:** Comparison of mean Euclidean distance error for the network architecture presented in this paper (EfficientNet B7/EfficientNet V2L + embeddings) and NetVLAD network for omnidirectional and panoramic images.

Experiment 2						
	**Configuration A**		**Configuration B**		**Configuration C**	
	**Omni**	**Panoramic**	**Omni**	**Panoramic**	**Omni**	**Panoramic**
**Neural Network**	$\bar{\bar{b_{L}}}$ **[m]**	$\bar{\bar{b_{L}}}$ **[m]**	$\bar{\bar{b_{L}}}$ **[m]**	$\bar{\bar{b_{L}}}$ **[m]**	$\bar{\bar{b_{L}}}$ **[m]**	$\bar{\bar{b_{L}}}$ **[m]**
EfficientNet B7 + embeddings	0.00	0.03	3.06	3.21	4.43	3.92
EfficientNet V2L + embeddings	0.00	0.00	2.34	3.11	4.94	3.60
NetVLAD (VGG16 + VLAD)	0.00	0.10	2.27	3.77	2.24	4.60

**Table 6:** Comparison of results for different approaches to place recognition (appearance-based localization) using images from a catadioptric camera. Best results shown in bold.

Global Descriptor	$\bar{\bar{b_{L}}}$ [m]	$\bar{\bar{b_{L}}}$ [m]	$\bar{\bar{b_{L}}}$ [m]
	Cloudy	Sunny	Night
EfficientNet V2L (training dataset 3)	0.22 ($\bar{\bar{t}}=0.32$ s)	**0.27** ($\bar{\bar{t}}=0.31$ s)	**0.20** ($\bar{\bar{t}}=0.31$ s)
EfficientNet V2L (training dataset 1)	0.24 ($\bar{\bar{t}}=0.32$ s)	0.44 ($\bar{\bar{t}}=0.32$ s)	0.33 ($\bar{\bar{t}}=0.32$ s)
CNN2 + FC6 [43]	0.29	0.69	0.29
CNN8 + FC6 [43]	0.25	0.93	0.24
HOG [32]	0.31	1.57	0.95
GIST [32]	**0.08**	1.23	1.31

## Data Availability

Open source code and our datasets of images are available on GitHub: https://github.com/mrostkowska/real-time-indoor-localization-catadioptric-vision-sensor.git, accessed on 18 June 2023. The used COLD dataset is available on this project’s web page (https://www.cas.kth.se/COLD/ accessed on 30 May 2023).
